# Assessing the Burden of Operatively Managed Extremity Fractures in Malawi: A Tale of Two Tertiary Hospitals

**DOI:** 10.1002/wjs.70475

**Published:** 2026-07-04

**Authors:** Cornelius Mukuzunga, Chikumbutso Clara Mpanga, Paul Chidothi, Esther Kawonga, Faith Moyo, Claude Martin, William J. Harrison

**Affiliations:** ^1^ Kamuzu Central Hospital (KCH) and Lilongwe Institute of Orthopaedics and Neurosurgery (LION) Trust Hospital Lilongwe Malawi; ^2^ Queen Elizabeth Central Hospital Blantyre Malawi; ^3^ Department of Surgery College of Medicine University of Malawi Blantyre Malawi; ^4^ Bradford University Hospital Bradford UK; ^5^ AO Alliance Foundation Chur Switzerland; ^6^ Countess of Chester NHS Foundation Trust Chester UK

**Keywords:** extremity fractures, low‐ and middle‐income countries, Malawi, motorcycle injuries, open fractures, trauma

## Abstract

**Background:**

Low‐ and middle‐income countries (LMICs) account for 90% of all deaths from injury globally, yet health systems remain poorly equipped to manage the escalating trauma burden.

**Purpose:**

This study describes the prevalence, causes, and surgical management of extremity fractures at Malawi's two high‐volume tertiary hospitals.

**Methods:**

A prospective cross‐sectional observational study collected injury mechanism and injury type as well as operative data on all patients undergoing surgical management of upper and lower extremity fractures at Queen Elizabeth Central Hospital (QECH) and Lilongwe Institute of Orthopedics and Neurosurgery (LION) Trust Hospital between January 2023 and March 2025. Descriptive statistics characterized study data, and ordered logistic regression evaluated factors associated with injury severity.

**Results:**

Details were recorded for 5674 patients and operations, comprising 79% males. Motorcycle collisions accounted for 35% of injuries, followed by falls (18%) and pedestrian‐related road traffic collisions (15%). One quarter of patients presented with open fractures, with higher proportions at QECH than at LION (30% vs. 22%, *p* = 0.001). The median time from admission to surgery was 7 days (interquartile range 1–18). Consultants performed 80% of surgeries at LION but only 16% at QECH (*p* < 0.001).

**Conclusions:**

This study highlights a critical and escalating operative trauma burden in Malawi, driven largely by road traffic injuries, particularly motorcycle taxis. The high prevalence of open fractures, surgical delays, and workforce disparities underscore the urgent need for coordinated health system reforms, including strengthening surgical infrastructure, expanding specialist training, and implementing evidence‐based road safety interventions.

**Level of Evidence:**

Level IV, Prognostic Study.

## Graphical abstract

Low‐ and middle‐income countries (LMICs) account for 90% of all deaths from injury globally, yet health systems remain poorly equipped to manage the escalating trauma burden. This study highlights a critical and escalating operative trauma burden in Malawi, driven largely by road traffic injuries, particularly motorcycle taxis. The high prevalence of open fractures, surgical delays, and workforce disparities underscore the urgent need for coordinated health system reforms, including strengthening surgical infrastructure, expanding specialist training, and implementing evidence‐based road safety interventions.

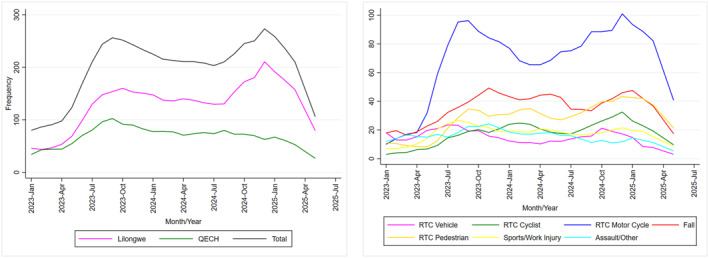

## 1 Introduction

The World Health Organization estimates that 4.4 million people die annually from injuries worldwide [1], with low‐ and middle‐income countries (LMICs) accounting for 90% of all deaths from injury [2]. Injuries have now surpassed HIV, tuberculosis, and malaria combined as the leading cause of death in LMICs [3]. Epidemiological data from LMICs indicate that injury prevalence is reaching epidemic proportions, with incident cases and severity continuing to rise [4]. A recent systematic review reported mean fracture incidence rates ranging from 779 to 1574 per 100,000 person‐years in LMICs [5], driven primarily by road traffic incidents, falls, and interpersonal assaults. Evidence also shows that for every death, 10 persons sustain permanent disability giving around 44 million new disabled persons per year, of which some 39.6 million reside in LMICs.

Although many injury‐related disabilities and deaths are preventable or treatable, health systems in LMICs remain poorly equipped to meet the growing demand for trauma care, leading to avoidable mortality and long‐term disability [1, 2]. Open fractures are among the most common types of injuries in LMICs and are known to carry significant risks of complications, including fracture‐related infection (18%), non‐union (15%), and amputation (15%) [6]. These complications often result in disability and impaired physical function, with substantial economic consequences for affected individuals. Evidence suggests that only one in five patients with open fractures return to work within one year of injury in LMICs [7] with the economic burden of injuries in LMICs exceeding $180 billion annually [8].

Malawi has the ninth highest rate of road traffic deaths globally, at 31 per 100,000 population, with 16% of all national fractures attributable to road traffic incidents [9]. Most road traffic‐related fractures and injuries in Malawi are associated with inadequate transport infrastructure and high‐risk modes of commuting, particularly motorcycle taxis [10]. Like most LMICs, Malawi faces a critical shortage of surgical infrastructure and workforce. Recent data indicate that Malawi has a specialist orthopedic physician‐to‐population ratio of 0.019 per 1000 people, substantially below the WHO recommendation of 2.5 per 1000 [11]. Consequently, task‐shifting is commonplace, with most district hospitals relying on clinical officers with limited training for orthopedic care. Additional health system inefficiencies that compromise the quality of injury and fracture care include delays in referral and treatment access. Only 63% of fracture patients at central hospitals and 47% at district hospitals receive referrals from lower‐level facilities for specialist care [8, 9, 10, 11]. Local efforts, largely supported by external funding, aim to develop guidelines and train clinical officers in trauma and orthopedic care, but systemic challenges persist [11, 12].

A recent study from Malawi assessed the fracture burden at district and central hospitals but provided limited insight into the severe fractures requiring surgical management [13]. In Malawi, all operative fracture care is delivered in four tertiary “central” hospitals, two of which are well established and perform the majority of the country's fracture surgeries. Evaluating the workload and injury patterns at these two centers therefore offers a meaningful national picture of severe extremity fractures which currently access surgical care. As the burden of injuries continues to rise across LMICs, there is an urgent need to better characterize the epidemiology of fractures to guide system planning. This paper describes the prevalence, causes, classification and surgical management of extremity fractures treated at the two high‐volume tertiary hospitals in Malawi between January 2023 and March 2025, to inform clinical practice, resource allocation, and national injury‐care policy in Malawi and other low and middle income countries.

## 2 Methods

### 2.1 Study Design and Setting

The study was conducted and reported in accordance with the Strengthening the Reporting of Observational Studies in Epidemiology (STROBE) guidelines for cross‐sectional studies. This prospective cross‐sectional observational study collected mechanism of injury, classification of injury, and operative data on patients undergoing surgical management of upper and lower extremity fractures in Malawi. The study was conducted at the two largest tertiary care public referral hospitals in Malawi: Queen Elizabeth Central Hospital (QECH) in Blantyre and Lilongwe Institute of Orthopedics and Neurosurgery (LION) Trust Hospital, located within Kamuzu Central Hospital in Lilongwe. QECH and LION are high‐volume hospitals serving a large urban and rural referral population of at least 15 million people, representing almost three quarters of Malawi's total population, with a combined bed capacity exceeding 2000. LION is a newly constructed hospital with five fully equipped operating theaters dedicated exclusively to trauma and orthopedic cases. QECH is an older facility dating to Malawi's colonial period, with two operating theaters dedicated to trauma and orthopedic cases.

This study was conducted during the AO Alliance Malawi Country Fracture Care Initiative and the Johnson & Johnson Long Bone Fracture Program. During this period, both hospitals benefited from substantial educational support and reliable access to essential surgical equipment. The AO Alliance is a non‐profit organization focused on strengthening fracture care in LMICs, and provides training, clinical mentorship, and systems‐level support to improve the delivery of safe and effective orthopedic trauma care.

### 2.2 Patient Recruitment

Participants were recruited consecutively between January 13, 2023, and March 31, 2025. Inclusion criteria comprised all patients who underwent surgical intervention for extremity fractures at either institution during the study period. All patients undergoing internal fixation or external fixation to stabilize their fractures were included. Patients who received non‐operative treatment modalities were excluded from this study.

### 2.3 Data Collection

Data collection occurred during the operative visit and included sociodemographic information (age, sex, occupation, educational level) and clinical information: (i) fracture characteristics (cause of injury, injury type, fracture classification, anatomic site involved); (ii) type of surgical procedure performed; (iii) grade of operating surgeon; and (iv) implant details. Standardized data collection forms ensured consistency across both sites. Trained research assistants, supervised by a clinical coordinator at each site, entered data into a secure electronic database. A random 10% sample of records was re‐abstracted and cross‐checked against the operative register to verify accuracy, and any discrepancies were reconciled before analysis. Because this was a consecutive, hospital‐wide census of all eligible operative cases over the study period, no prior sample size was calculated.

### 2.4 Statistical Analysis

Descriptive statistics were used to characterize the study population and injury patterns at the two hospitals. Proportions were compared using Pearson's chi‐squared test, whereas the Wilcoxon rank‐sum test and Kruskal‐Wallis test compared medians of continuous variables. An ordered logistic regression model was used to evaluate how management approaches changed with increasing severity among patients. Injury severity, defined using the AO/OTA fracture classification, served as the ordinal outcome; demographic, anatomical, and management covariates were modeled as independent variables, and the proportional‐odds assumption was tested using the Brant test. Two‐sided *p*‐values less than 0.05 were considered statistically significant, and 95% confidence intervals are reported where appropriate. Missing data were handled through complete‐case analysis, and the proportion of missing observations is reported for each variable in the corresponding table. All analyses were conducted using Stata statistical software (StataCorp, College Station, TX, USA).

Ethics clearance was obtained from the Kamuzu University of Health Sciences Institutional Review Board (COMREC approval number P.08/23‐0188). No patient‐traceable data was recorded in the study database.

## 3 Results

### 3.1 Profile of the Study Population

Details were recorded for 5674 surgical episodes, comprising 79% males. The majority were from LION Trust Hospital (66%) (Table 1). At baseline, the prevalence of recorded comorbid conditions, including HIV, hypertension, diabetes, and epilepsy, was low, affecting 245 of 5674 participants (4%).

**TABLE 1:** Sociodemographic characteristics of study participants by hospital.

Characteristic	Total *N* = 5674	Lilongwe *n* = 3728	QECH *n* = 1946
Age (Years)			
Median (IQR)	34 (23–45)	35 (24–45)	33 (23–44)
< 18	737 (13.0%)	470 (12.6%)	267 (13.7%)
18–29	1504 (26.5%)	957 (25.7%)	547 (28.1%)
30–39	1313 (23.1%)	871 (23.4%)	442 (22.7%)
40–49	1066 (18.8%)	706 (18.9%)	360 (18.5%)
50–59	573 (10.1%)	383 (10.3%)	190 (9.8%)
60+	473 (8.3%)	340 (9.1%)	133 (6.8%)
Missing	8 (0.1%)	1 (0.0%)	7 (0.4%)
Sex			
Males	4444 (78.3%)	3000 (80.5%)	1444 (78.3%)
Patient occupation			
Small business	1908 (33.6%)	1472 (39.5%)	436 (22.4%)
Farmer	1286 (22.7%)	1040 (27.9%)	246 (12.6%)
Construction/manual labor	821 (14.5%)	410 (11.0%)	411 (21.1%)
Child/Student	737 (13.0%)	470 (12.6%)	267 (13.7%)
Civil service/Professional	546 (9.6%)	245 (6.6%)	301 (15.5%)
Unemployed	340 (6.0%)	91 (2.4%)	249 (12.8%)
Missing	36 (0.6%)	0 (0.0%)	36 (1.9%)
Education			
No formal education	2208 (38.9%)	1340 (35.9%)	868 (44.6%)
Jnr secondary certificate	1367 (24.1%)	1114 (29.9%)	253 (13.0%)
Snr secondary certificate	1059 (18.7%)	749 (20.1%)	310 (15.9%)
Secondary	358 (6.3%)	141 (3.8%)	217 (11.2%)
Primary	637 (11.2%)	384 (10.3%)	253 (13.0%)
Smoking status			
Smokers	293 (5.2%)	184 (4.9%)	109 (5.6%)
HIV status			
Seropositive	144 (2.5%)	16 (0.4%)	128 (6.6%)
Diabetes status			
Had diabetes	17 (0.3%)	4 (0.1%)	13 (0.7%)
Epilepsy			
Had epilepsy	18 (0.3%)	9 (0.2%)	9 (0.5%)
Comorbidities			
Single conditions	230 (4.1%)	21 (0.6%)	209 (10.7%)
Two or more conditions	15 (0.3%)	1 (0.0%)	14 (0.7%)
Cause of injury			
RTC motorcycle	1974 (34.8%)	1546 (41.5%)	428 (22.0%)
Fall	1005 (17.7%)	451 (12.1%)	554 (28.5%)
RTC pedestrian	808 (14.2%)	457 (12.3%)	351 (18.0%)
RTC cyclist	510 (9.0%)	469 (12.6%)	41 (2.1%)
Sports/Work injury	499 (8.8%)	382 (10.3%)	117 (6.0%)
Assault/Other	438 (7.7%)	153 (4.1%)	285 (14.7%)
RTC vehicle	416 (7.3%)	270 (7.2%)	146 (7.5%)
Missing	24 (0.4%)	0 (0.0%)	24 (1.2%)

Abbreviation: RTC = Road traffic collision.

Nearly 40% of participants reported no formal education, with a higher proportion at QECH (45%). Children aged 16 years and under accounted for 12% of the study population (*n* = 650), with similar proportions at both hospitals (11% at Lilongwe and 13% at QECH). Table 1 describes the sociodemographic profile of the study population.

### 3.2 Causes of Injury

**FIGURE 1:**
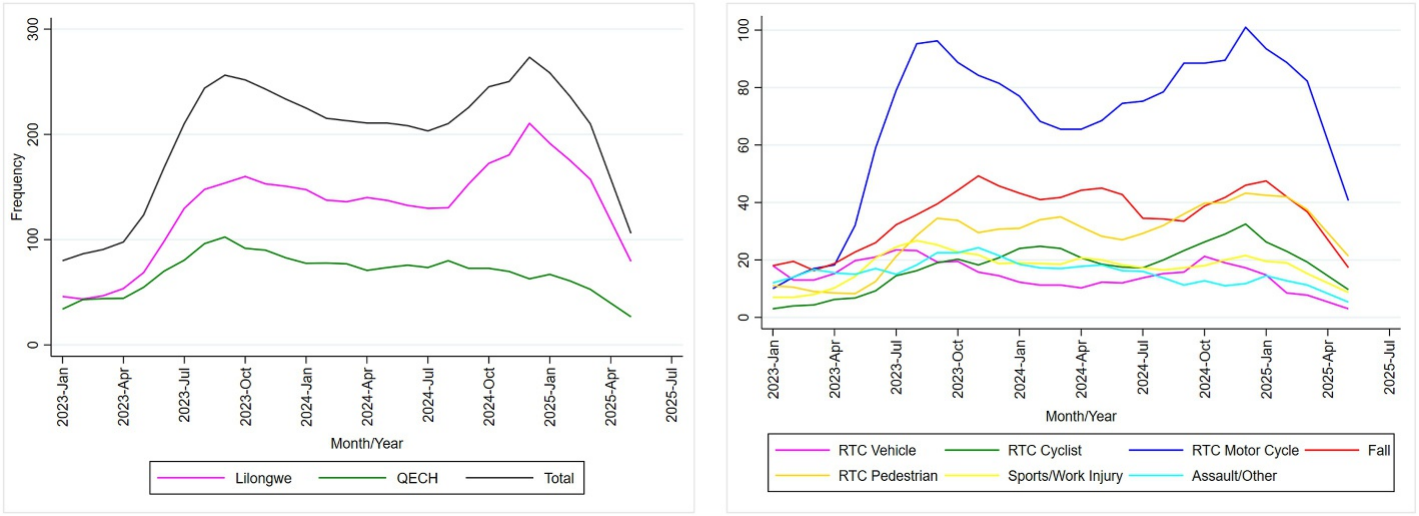
(a) Distribution of injury causes among study participants. (b) Trends in injury causes over the study period (January 2023 to March 2025).

Motorcycle collisions accounted for a substantial proportion of injuries in the study (35%), followed by falls (18%) and pedestrian‐related road traffic collisions (15%). The remaining injuries were attributable to vehicle collisions, cycling accidents, sports or work‐related injuries, and assaults, each comprising less than 10% of cases (Figure 1a and b). During the study period, significant increases in injuries from motorcycles, falls, and pedestrian‐related incidents were observed, with trends being statistically significant in each case (*p* < 0.001) (Figure 1b). Causes of injury were significantly associated with participants' age and sex in each case (*p* < 0.001) (Supporting Information S1: Table 1). Among participants aged 16 years and older with documented AO fracture classification (*n* = 4749), motorcycle injuries accounted for 790 of 1957 (41%) of AO classification grade B, and 478 of 1249 (38%) of AO grade C injuries, respectively.

### 3.3 Clinical Characteristics of Fractures and Management

**FIGURE 2:**
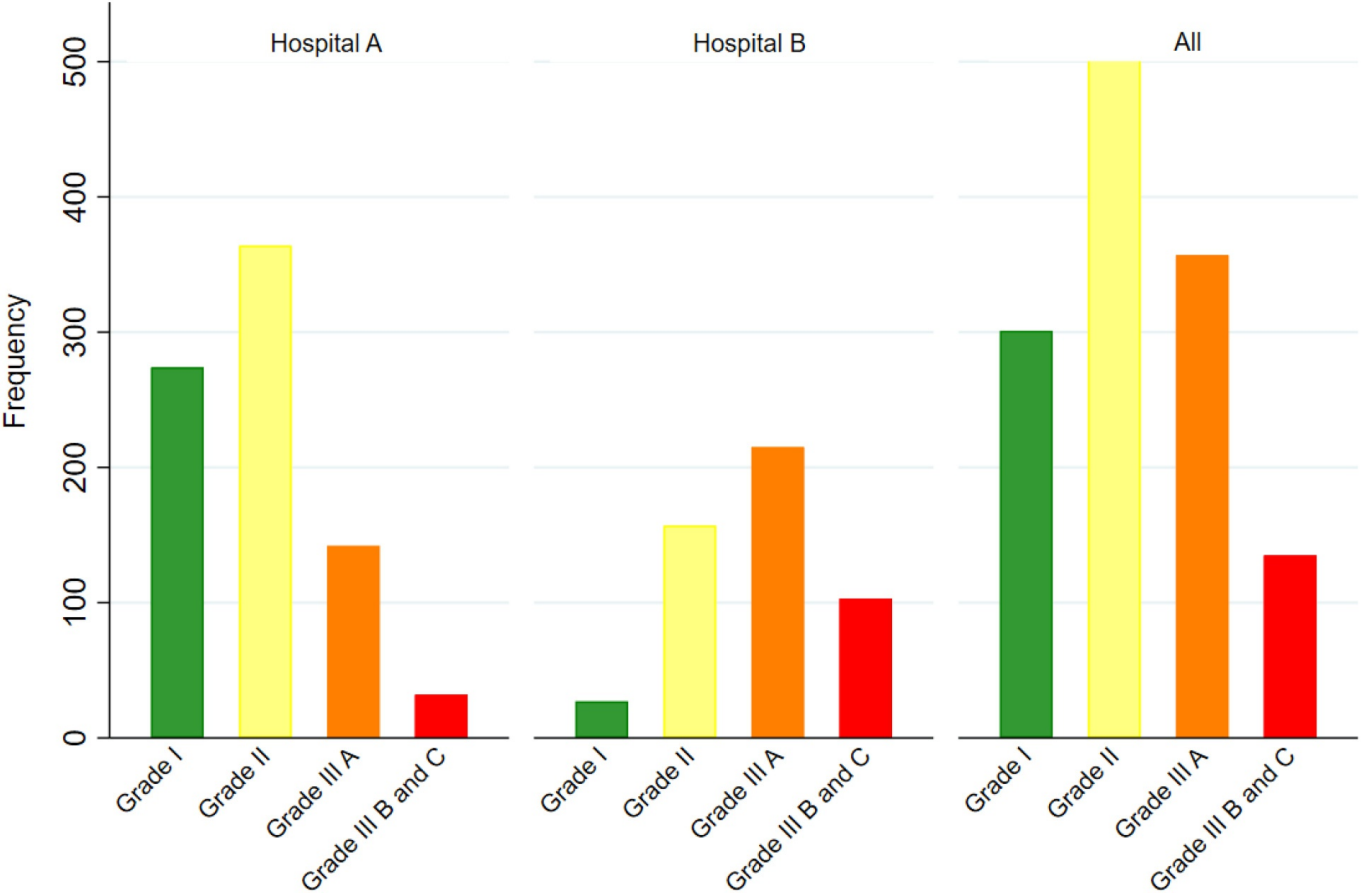
Distribution of Gustilo classification grades for open fractures at QECH and LION Trust Hospital.

One quarter of the study population presented with open fractures overall, with higher proportions observed at QECH compared to Lilongwe (30% vs. 22%, *p* = 0.001) (Table 2). Furthermore, 68% of open fractures at QECH were classified as Gustilo Grade 3B/C, compared to 32% at Lilongwe Hospital (*p* < 0.001) (Figure 2). Among participants (aged 16 years and older) with documented AO fracture classification (*n* = 4749), two‐thirds had an AO classification of Grade B or C, with proportions of 68% at Lilongwe and 65% at QECH (Table 2).

**TABLE 2:** Clinical characteristics of presenting fractures and surgical procedures performed in Malawi, 2023–2025.

Characteristic	Total *N* = 5674	Lilongwe *n* = 3728	QECH *n* = 1946	*p* value
Injury type				**< 0.001**
Single	4615 (81.3%)	3236 (86.8%)	1379 (71.0%)	
Poly trauma	1051 (18.5%)	492 (13.2%)	559 (28.7%)	
Missing	8 (0.1%)	0 (0.0%)	8 (0.4%)	
Fracture classification				**0.001**
Open fractures	1395 (24.6%)	821 (22.0%)	574 (29.5%)	
Closed fractures	4279 (75.4%)	2907 (78.0%)	1372 (70.5%)	
Fracture type				**0.001**
Acute	5443 (95.9%)	3607 (97.8%)	1836 (94.4%)	
Trauma sequelae	84 (1.5%)	33 (0.9%)	51 (2.6%)	
Trauma consequences	102 (1.8%)	55 (1.5%)	47 (2.4%)	
Missing	45 (0.8%)	33 (0.9%)	12 (0.6%)	
Injury AO classification (> 16 years)				**< 0.001**
A	1542 (32.5%)	1039 (31.3%)	503 (35.1%)	
B	1958 (41.2%)	1499 (45.2%)	459 (32.0%)	
C	1249 (26.3%)	778 (23.5%)	471 (32.9%)	
Anatomic site				**0.001**
Tibia/Fibula	1381 (24.3%)	890 (23.9%)	491 (25.2%)	
Distal	283 (20.5%)	180 (20.2%)	103 (21.0%)	
Midshaft	663 (48.0%)	441 (49.6%)	222 (45.2%)	
Proximal	435 (31.5%)	269 (30.2%)	166 (33.8%)	
Radius/Ulna	1066 (18.8%)	748 (20.1%)	318 (16.3%)	
Distal	418 (39.2%)	308 (41.2%)	110 (34.6%)	
Midshaft	277 (26.0%)	154 (20.6%)	123 (38.7%)	
Proximal	371 (34.8%)	286 (38.2%)	85 (26.7%)	
Femur	1332 (23.4%)	949 (25.5%)	383 (19.7%)	
Distal	468 (35.1%)	312 (32.9%)	156 (40.7%)	
Midshaft	641 (48.1%)	487 (51.3%)	154 (40.2%)	
Proximal	223 (16.7%)	150 (15.8%)	73 (19.1%)	
Humerus	568 (10.0%)	262 (7.0%)	306 (15.7%)	
Distal	474 (83.5%)	199 (76.0%)	275 (90.0%)	
Midshaft	68 (12.0%)	42 (16.0%)	26 (8.5%)	
Proximal	26 (4.6%)	21 (8.0%)	5 (1.6%)	
Ankle	765 (13.5%)	523 (14.0%)	242 (12.4%)	
Pelvis	188 (3.3%)	119 (3.2%)	69 (3.6%)	
Patella	159 (2.8%)	111 (3.0%)	48 (2.5%)	
Other	215 (3.8%)	126 (3.4%)	89 (4.6%)	
Surgical procedure				**0.001**
Fracture external fixation	857 (15.1%)	460 (12.3%)	397 (20.4%)	
External fixation monoplanar	853 (99.5%)	460 (100.0%)	393 (99.0%)	
External fixation circular	4 (0.5%)	0 (0.0%)	4 (1.0%)	
Fracture internal fixation	4812 (84.9%)	3268 (87.7%)	1544 (79.6%)	
ORIf plate	2767 (57.5%)	1930 (59.1%)	837 (54.2%)	
ORIf nail	1092 (22.7%)	721 (22.1%)	371 (24.0%)	
ORIF K‐wire	524 (10.9%)	333 (10.2%)	191 (12.4%)	
ORIf tension band wire	164 (3.1%)	114 (3.5%)	50 (3.2%)	
ORIf special plate	115 (2.4%)	101 (3.1%)	14 (0.9%)	
CRIF K‐wire	112 (2.3%)	60 (1.8%)	52 (3.4%)	
Other	8 (0.2%)	5 (0.2%)	3 (0.2%)	
Timing of operative procedure				**< 0.001**
Primary procedure	5525 (97.4%)	3662 (98.2%)	1863 (95.7%)	
Secondary procedure	149 (2.6%)	66 (1.8%)	83 (4.3%)	
Grade of surgeon				**0.001**
Consultant	3316 (58.4%)	2996 (80.4%)	320 (16.4%)	
Resident	2191 (38.6%)	677 (18.2%)	1514 (77.8%)	
Orthopedic clinical officer	150 (2.6%)	44 (1.2%)	106 (5.5%)	
Other	17 (0.3%)	11 (0.3%)	6 (0.3%)	

*Note:* The bold *p* values are denoting to statistically significant at 5%.

Abbreviations: CRIF = Closed Reduction Internal Fixation, ORIF = Open Reduction Internal Fixation.

Among participants aged 16 years and older, surgically treated injuries predominantly affected the lower limb (midshaft or proximal tibia/fibula and the distal or midshaft femur) (Supporting Information S1: Figure 1). Among children (≤ 16 years) with documented injury site and limb involvement (*n* = 650), the most common surgically treated fractures involved the upper limb (distal humerus and the proximal radius/ulna). In bivariate analyses, the participants' age group, sex, and cause of injury were each significantly associated with both the location of injury and the limb involved (*p* < 0.001 for all; Table 3).

**TABLE 3:** Classification of fracture types by age group, sex and cause of injury.

	Anatomic site								
	Radius/Ulna *n* = 1066	Tibia/Fibula *n* = 1381	Humerus *n* = 568	Femur *n* = 1332	Ankle *n* = 765	Pelvis *n* = 188	Patella *n* = 159	Other *n* = 215	Total *N* = 5674
Age group									***p* < 0.001**
< 18	201 (18.9%)	80 (5.8%)	214 (37.7%)	194 (14.6%)	19 (2.5%)	9 (4.8%)	3 (1.9%)	17 (7.9%)	737 (13.0%)
18–29	283 (26.6%)	408 (29.6%)	120 (21.2%)	372 (28.0%)	155 (20.3%)	45 (24.1%)	39 (24.5%)	82 (38.1%)	1504 (26.5%)
30–39	244 (22.9%)	338 (24.5%)	90 (15.9%)	272 (20.4%)	211 (27.7%)	62 (33.2%)	43 (27.0%)	53 (24.7%)	1313 (23.2%)
40–49	174 (16.3%)	302 (21.9%)	69 (12.2%)	206 (15.5%)	212 (27.8%)	33 (17.6%)	30 (18.9%)	40 (18.6%)	1066 (18.8%)
50–59	89 (8.4%)	128 (9.3%)	40 (7.1%)	142 (10.7%)	103 (13.5%)	33 (17.7%)	22 (13.8%)	16 (7.4%)	573 (10.1%)
60+	75 (7.0%)	123 (8.9%)	34 (6.0%)	145 (10.9%)	62 (8.1%)	5 (2.3%)	22 (13.8%)	7 (3.3%)	473 (8.4%)
Sex									***p* < 0.001**
Female	194 (18.2%)	233 (16.9%)	163 28.8%)	292 21.9%)	242 (31.6%)	45 (23.9%)	22 (13.8%)	37 (17.2%)	1228 (21.7%)
Male	871 (81.8%)	1148 (83.1%)	404 (71.3%)	1040 (78.1%)	523 (68.4%)	143 (76.1%)	137 (86.2%)	178 (82.8%)	4444 (78.4%)
Cause of injury									***p* < 0.001**
RTC vehicle	134 (6.3%)	244 (8.9%)	38 (3.4%)	228 (8.6%)	74 (4.8%)	66 (17.7%)	24 (7.6%)	24 (7.6%)	832 (7.4%)
RTC cyclist	192 (9.0%)	236 (8.6%)	70 (6.2%)	256 (9.7%)	144 (9.4%)	38 (10.2%)	42 (13.2%)	42 (9.8%)	1020 (9.0%)
RTC motorcycle	672 (31.6%)	1120 (40.9%)	256 (22.6%)	1014 (38.3%)	518 (33.9%)	144 (38.5%)	104 (32.7%)	120 (28.0%)	3948 (34.9%)
Fall	376 (17.7%)	228 (8.3%)	462 (40.7%)	400 (15.1%)	398 (26.1%)	30 (8.0%)	72 (22.6%)	44 (10.3%)	2010 (17.8%)
RTC pedestrian	240 (11.3%)	528 (19.3%)	104 (9.2%)	354 (13.4%)	242 (15.8%)	56 (15.0%)	32 (10.1%)	60 (14.0%)	1616 (14.3%)
Sports/work	290 (13.6%)	158 (5.8%)	106 (9.4%)	266 (10.1%)	78 (5.1%)	22 (5.9%)	22 (6.9%)	56 (13.1%)	998 (8.8%)
Assault/other	226 (10.6%)	228 (8.3%)	98 (8.6%)	128 (4.8%)	74 (4.8%)	18 (4.8%)	22 (6.9%)	82 (19.2%)	876 (7.8%)

*Note:* The bold *p* values are denoting to statistically significant at 5%.

Abbreviation: RTC = road traffic collision, proportions based on non‐missing values.

### 3.4 Clinical Management of Fractures

**FIGURE 3:**
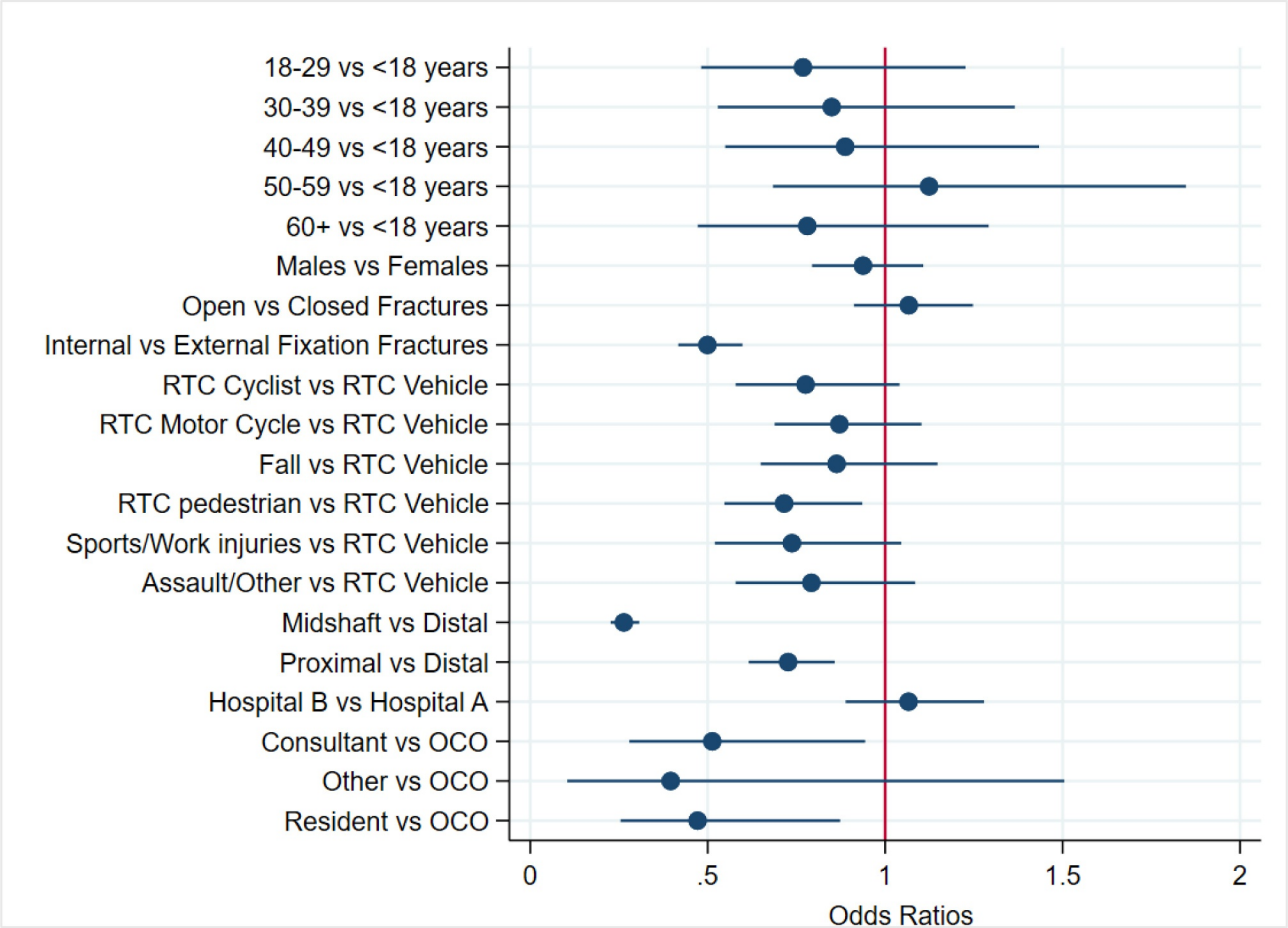
Forest plot showing results of ordered logistic regression model examining factors associated with injury severity.

Overall, 58% of cases were managed by trauma and orthopedic consultants and 39% by resident surgeons. The grade of operating surgeon differed significantly between the two hospitals, with 80% of surgeries performed by consultants at Lilongwe compared to 16% at QECH. The overall median time (interquartile range) from admission to surgery was 7 days (1–18), with similar values at both hospitals (7 days [0–19] at Lilongwe and 7 days [2–14] at QECH) (Supporting Information S1: Figure 2). Time from admission to surgery varied significantly by severity of injury (AO classification, *p* = 0.03), between males and females (*p* < 0.001), grade of the surgeon (*p* = 0.002), and cause of injury (*p* < 0.001). The ordered logistic regression analysis showed that the likelihood of presenting with a more severe injury was lower (i) among patients with mid‐shaft or proximal fractures compared with distal fractures, (ii) among those treated with external rather than internal fixation, and (iii) among pedestrians compared with occupants of motor vehicles (Figure 3). For other variables, the statistical evidence was less certain; injury‐severity management did not differ by age group, fracture type (open vs. closed), hospital site, or between motorcycle‐related and other motor‐vehicle‐related road traffic collisions.

## 4 Discussion

This large prospective cross‐sectional study of injury data among patients undergoing surgical management of extremity fractures in Malawi reveals a substantial and growing operative trauma burden, driven primarily by motorcycle collisions and pedestrian road traffic incidents. In this study, young adult males were disproportionately affected, and injury patterns varied significantly by age and anatomical site. A high prevalence of open fractures was observed, with significantly higher rates and severity at QECH versus LION Hospital. Marked workforce disparities between the two hospitals were evident, with consultants performing 80% of surgeries at Lilongwe but only 16% at QECH.

Our findings align with evidence from similar studies in LMICs [14]. The predominance of road traffic injuries (vehicles, cyclists, and motorcycles combined) is consistent with WHO reports identifying these as the leading cause of injury‐related disability in LMICs [15, 16]. However, the specific dominance of motorcycle collisions exceeds rates reported in many African trauma studies. This likely reflects Malawi's mounting transport challenges, including the use of motorcycles as taxis, inadequate public transport and weak enforcement of traffic regulations. Furthermore, the prevalence of open fractures is substantially higher than in high‐income countries (typically 5%–15%) providing evidence that LMICs bear a disproportionate burden of severe injuries [17, 18]. The use of motorcycle taxis which has largely arisen in the last 10 years in Malawi, and observed during this short period of this study, has altered the number and severity of fractures requiring operative management. It also parallels a steep rise in prevalence of open fractures. This serves as a warning to other countries who may wish to adopt this transport strategy and highlights a pressing need for improved regulation in Malawi. In the local context, the higher open fracture burden at QECH compared to LION may be explained by the historical context that Blantyre is an older city with cramped road networks while Lilongwe, the capital city, is a larger and more open city with bigger roads.

The 7‐day median surgical delay contrasts sharply with high‐income country standards (< 24 h for open fractures) but aligns with data from other resource‐constrained settings such as Tanzania and Uganda, where similar systemic delays are documented [19, 20]. Contributing factors include specialist shortages, operating theater constraints. Patients in Malawi are not required to pay for their implants, but this process is known to cause further delays in many African health systems [21, 22]. The stark workforce disparity between the two hospitals likely stems from historical resource allocation patterns and external training partnerships concentrated in Lilongwe. This may also have been impacted by may also be impacted by the private‐public partnership model adopted at LION Hospital. The association between external fixation and more severe injuries in our regression model suggests its use as a damage‐control strategy in resource‐limited settings rather than as a definitive treatment solution.

A substantial proportion of operations were performed by resident surgeons operating without a consultant scrubbed, most strikingly at QECH, where consultants were present for only 16% of procedures compared with 80% at LION. Within the Malawian training and regulatory framework, this is an accepted and long‐standing practice: senior registrars and medical officers registered with the Medical Council of Malawi are licensed to undertake operative fracture care within their scope of competence, with a consultant available on call for consultation and escalation rather than necessarily present in theater. This model of supervised independence is the norm across much of sub‐Saharan Africa and reflects a necessary adaptation to severe specialist shortages; it differs from practice in many high‐income settings, such as the United States, where attending‐surgeon presence is generally required for operative cases. While such delegated operating expands surgical capacity and is sanctioned within local accreditation and licensing structures, it warrants explicit acknowledgment here, particularly because the time from admission to surgery differed significantly by grade of operating surgeon (*p* = 0.002). The greater reliance on resident‐led operating at QECH may reflect both its more acute workforce constraints and the concentration of consultant staffing at LION. These observations reinforce the importance of structured supervision, mentorship, and clearly defined escalation pathways to safeguard operative quality and patient safety as task‐sharing is scaled up.

Our findings have important implications for national and regional policy. To reduce road traffic collisions and injuries, policy initiatives should prioritize road safety. Targeted interventions may include motorcycle safety measures especially where motorcycles are used as taxis and pedestrian protection through improved road infrastructure. For health systems, scaling up trauma and orthopaedic specialist training programs and standardizing trauma care protocols for task‐sharing clinicians may improve the quality of clinical care and patient outcomes. This will require strategic allocation of healthcare resources, with priority investment in surgical infrastructure (operating theatres and implant supply chains) to reduce time to surgery. Referral systems also require strengthening, beginning with robust pre‐hospital care and inter‐facility referral networks to ensure timely access to appropriate care and return to local hospitals for rehabilitation at lower cost and nearer to home [23, 24].

Strengths of this study include the large prospective sample from Malawi's two major tertiary centres with standardized data collection, the use of internationally recognised fracture classification systems (AO/OTA and Gustilo–Anderson), which enhances comparability with international cohorts. To our knowledge, this is the largest study of its type from a low‐ and middle‐income country and it builds on the previous contextual study describing non‐operative care in the Malawi health care system. Limitations include the specific operative‐based analysis chosen which does not allow evaluation of the complete fracture burden and notably omits cases which would have benefitted from surgery but did not reach these treating facilities. Because only surgically treated patients were included, selection bias is possible; patients who died before reaching a tertiary centre, those managed non‐operatively, and those who self‐discharged are not represented. The cross‐sectional design limits causal inference, and we lack outcome data on infection rates, functional recovery, and return to work. Residual confounding by unmeasured variables (such as injury mechanism severity, pre‐hospital transport time, and comorbidities beyond those recorded) may influence the regression estimates. We also noted an apparent fall in recorded injuries towards the very end of the study period (Figure 1b). We interpret this cautiously as most likely an artefact of the data‐capture process rather than a true reduction in injury incidence: cases operated in the final weeks were more susceptible to incomplete ascertainment and delayed data entry, and the consecutive‐census design left these late records less time to be reconciled against the operative register before the analysis cut‐off. A genuine decline over this short interval is unlikely given the significant upward trends observed for motorcycle, fall, and pedestrian‐related injuries across the period, and this end‐of‐study tail should therefore be regarded as a recording artefact rather than an epidemiological trend. Finally, because the two participating hospitals benefited from concurrent educational and equipment support through the AO Alliance and the Johnson & Johnson Long Bone Fracture Programme, the patterns of care described may not generalise to other low‐resource settings without comparable support.

In conclusion, this study highlights a critical trauma burden in Malawi, driven largely by road traffic injuries and compounded by systemic healthcare challenges. The high prevalence of open fractures, surgical delays, and limited workforce underscore the urgent need for health system reforms. Strengthening surgical infrastructure, expanding specialist training, and improving referral pathways will be essential to addressing the trauma burden in Malawi. Policy interventions focused on road safety, in particular related to motorcycle taxis, and injury prevention will be critical to reducing the incidence of severe trauma. Future research should prospectively link these operative data to longitudinal outcomes, including fracture‐related infection, union, functional recovery, and return to work, and should extend surveillance to district hospitals to capture the full spectrum of fracture care across Malawi.

## Author Contributions

**Cornelius Mukuzunga:** conceptualization, methodology, data curation, investigation, validation, formal analysis, supervision, project administration, writing ‐ review and editing, writing ‐ original draft. **Chikumbutso Clara Mpanga:** conceptualization, methodology, data curation, investigation, validation, formal analysis, supervision, project administration, writing ‐ review and editing. **Paul Chidothi:** investigation, validation, project administration, writing ‐ review and editing, data curation, supervision. **Esther Kawonga:** investigation, validation, supervision, data curation, writing ‐ review and editing. **Faith Moyo:** methodology, data curation, formal analysis, writing ‐ original draft, writing ‐ review and editing, software. **Claude Martin Jr:** conceptualization, methodology, data curation, formal analysis, supervision, resources, project administration, funding acquisition, writing ‐ review and editing, software. **William J. Harrison:** conceptualization, methodology, software, data curation, validation, funding acquisition, writing ‐ review and editing, resources, visualization.

## Funding

This work was supported by equipment and funding from the Johnson & Johnson Global Surgery grant for the “Malawi Long Bone Fracture Program.” The funder had no role in study design, data collection, analysis, interpretation, manuscript preparation, or the decision to submit for publication.

## Ethics Statement

Ethics clearance was obtained from the Kamuzu University of Health Sciences Institutional Review Board (COMREC approval number P.08/23‐0188).

## Consent

Because the study used routinely collected, de‐identified operative data, the requirement for individual informed consent was waived by the review board. All procedures were performed in accordance with the ethical standards of the 1964 Declaration of Helsinki and its later amendments.

## Conflicts of Interest

The authors declare no conflicts of interest.

## Supporting information


Supporting Information S1

## Data Availability

The de‐identified dataset that supports the findings of this study is available from the corresponding author upon reasonable request, subject to the approval of the Kamuzu University of Health Sciences Institutional Review Board and the participating hospitals.
