# Supplementary material for: Assessing the Burden of Operatively Managed Extremity Fractures in Malawi: A Tale of Two Tertiary Hospitals
Source: World J Surg. 2026 Jul 4;50(8):2480–90. doi: 10.1002/wjs.70475 (PMC13460881; doi:10.1002/wjs.70475)
Supplement: Supplementary file 1 [file WJS-50-2480-s001.docx]

Supplementary Table 1. Causes of injury stratified by age groups and sex of participants

| **Age group** | **Sex** | **Total N=5650** | **RTC**  **Vehicle n=416** | **RTC**  **Cyclist n=510** | **RTC**  **Motorcycle n=1 974** | **Fall n=1 005** | **RTC**  **Pedestrian n=808** | **Sports/ Work injury n=499** | **Assault/ Other n=438** |
| --- | --- | --- | --- | --- | --- | --- | --- | --- | --- |
| **<18** | Female | 175 | 5 (2.9%) | 5 (2.9%) | 22 (12.6%) | 59 (33.7%) | 18 (10.3%) | 58 (33.1%) | 8 (4.6%) |
|  | Male | 560 | 14 (2.5%) | 38 (6.8%) | 56 (10.0%) | 213 (38.0%) | 35 (6.3%) | 183 (32.7%) | 21 (3.8%) |
|  | |  |  | | | | | | |
| **18-29** | Female | 302 | 41 (13.6%) | 18 (6.0%) | 86 (28.5%) | 69 (22.9%) | 46 (15.2%) | 9 (3.0%) | 33 (10.9%) |
|  | Male | 1196 | 65 (5.4%) | 109 (9.1%) | 521 (43.6%) | 88 (7.4%) | 173 (14.5%) | 151 (12.6%) | 89 (7.4%) |
|  | |  |  | | | | | | |
| **30-39** | Female | 243 | 32 (13.2%) | 6 (2.5%) | 86 (35.4%) | 65 (26.8%) | 29 (11.9%) | 5 (2.1%) | 20 (8.2%) |
|  | Male | 1065 | 101 (9.5%) | 117 (11.0%) | 464 (43.6%) | 95 (8.9%) | 162 (15.2%) | 41 (3.9%) | 85 (8.0%) |
|  | |  |  | | | | | | |
| **40-49** | Female | 188 | 15 (8.0%) | 10 (5.3%) | 57 (30.3%) | 65 (34.6%) | 31 (16.5%) | 2 (1.1%) | 8 (4.3%) |
|  | Male | 874 | 71 (8.1%) | 113 (12.9%) | 343 (39.2%) | 86 (9.8%) | 154 (17.6%) | 23 (2.6%) | 84 (9.6%) |
|  | |  |  | | | | | | |
| **50-59** | Female | 153 | 17 (11.1%) | 7 (4.6%) | 53 (34.6%) | 48 (31.4%) | 15 (9.8%) | 1 (0.7%) | 12 (7.8%) |
|  | Male | 417 | 34 (8.2%) | 52 (12.5%) | 168 (40.3%) | 38 (9.1%) | 67 (16.1%) | 17 (4.1%) | 41 (9.8%) |
|  | |  |  | | | | | | |
| **60+** | Female | 158 | 6 (3.8%) | 6 (3.8%) | 39 (24.7%) | 79 (50.0%) | 18 (11.4%) | 3 (1.9%) | 7 (4.4%) |
|  | Male | 312 | 15 (4.8%) | 29 (9.3%) | 79 (25.3%) | 95 (30.5%) | 59 (18.9%) | 5 (1.6%) | 30 (9.6%) |

Proportions based on non-missing values

1

Supplementary Figure 1. Proportion of injuries by anatomic site and limb type between children and adults

Supplementary Figure 2. Time from presentation to surgery stratified by year and health facility.
